# “Living like I am in Thailand”: stress and coping strategies among Thai migrant masseuses in Oslo, Norway

**DOI:** 10.1186/s12905-019-0836-9

**Published:** 2019-11-15

**Authors:** Naomi Tschirhart, Melanie Straiton, Trygve Ottersen, Andrea S. Winkler

**Affiliations:** 1 Oslo Group on Global Health Policy, Department of Community Medicine and Global Health and Centre for Global Health, Institute of Health and Society, Faculty of Medicine, University of Oslo, Postboks 1130 Blindern, 0318 Oslo, Norway; 2 Department for Mental Health and Suicide, Norwegian Institute of Public Health, PO Box 222 Skøyen, 0213 Oslo, Norway; 3 Division for Health Services, Norwegian Institute of Public Health, PO Box 222 Skøyen, 0213 Oslo, Norway; 4 Centre for Global Health, Institute of Health and Society, University of Oslo, Postboks 1130 Blindern, 0318 Oslo, Norway; 5 Department of Neurology, Center for Global Health, Technical University of Munich, Ismaninger Straße 22, 81675 Munich, Germany

**Keywords:** Thai, Migrant, Masseuse, Marriage, Mental health, Wellbeing, Stress, Coping

## Abstract

**Background:**

Migrants experience stress before, during and after migrating to a new country, all of which influences their mental wellbeing. In Norway, migration from Thailand is highly gendered as most Thai migrants are women who migrate to live with their Norwegian spouse. Massage shops, often owned by Thai entrepreneurs, are a locale where women use their cultural knowledge to bridge into the local economy. There is little knowledge about Thai migrant masseuses’ experience of stress in daily life and associated coping strategies. The objective of this inquiry was to examine stressors and coping strategies among Thai migrant masseuses in Oslo, Norway.

**Methods:**

We conducted in-depth interviews with 14 Thai migrants who were working as masseuses in Oslo, Norway. We asked participants about their health, experiences of stress, and coping strategies and subsequently analyzed the data using thematic analysis.

**Results:**

Stress in participants’ lives related to settling in, loneliness, finances and spousal relationships. Of these, relationship conflict was the largest source of stress. Women largely embraced self-coping strategies and utilized Thai cultural practices and Buddhist cognitive thinking. Once relationship conflict became untenable, participants fought to change their situation. Limited fluency in Norwegian, Thai stigma about mental health and limited knowledge of the Norwegian health system were barriers to seeking healthcare.

**Conclusions:**

Migrants in our study often adopted “Thainess”, the use of Thai cultural practices and Buddhist cognitive thinking, as a strategy for coping with stress. Preferences for self-coping, mental health stigma, and linguistic competency are important considerations when designing mental wellbeing interventions for Thai women. Use of an interpreter or systems navigator can help overcome language barriers. Clinicians can take detailed case histories to better understand Thai patients’ stress, coping strategies and wellbeing. Health policy makers could consider network approaches, including using Thai health systems navigators to bridge the health system and Thai communities.

## Background

Globally, 244 million people are international migrants living outside of their country of birth [1]. Migrants experience stressors before, during migration and while living in the destination country, all of which influence their mental health and wellbeing [2–4]. Mental health is also influenced by the socio-economic factors and supports that are available. Individuals who experienced a voluntary planned migration, and have sufficient language proficiency and social support in their new country are less likely to experience common mental disorders than others who had forced or precarious journeys [5]. Stress experienced by migrants and coping strategies differs between cultural groups [3]. Research on how different groups experience and cope with stress can be used to support interventions to improve migrants’ wellbeing. This study explores the stress and coping experiences of Thai migrant masseuses.

**Table 1:** Demographic characteristics of interview participants (*n* = 14)

Characteristics	N
Age	
< 34–39	3
40–45	4
> 45	7
Years in Norway	
< 2	5
3–10	4
> 10	5
Fluency in Norwegian	
Limited	3
Beginner	7
Intermediate	3
Not stated	1
Marital Status	
Married/Cohabitating	2
Separated/Divorced	9
Widowed	1
Single	2
Monthly Income in Norwegian Kroner (NOK)	
< 15,000	2
16,000-20,000	5
21,000-25,000	0
26,000-30,000	1
> 31,000	5
Not stated	1

As a result of migration, Norway’s population is becoming more diverse. Migration flows from Thailand to Norway have been increasing and Thai women are the largest group that come to Norway through the family migration channel as the spouse of a Norwegian husband [6, 7]. This reflects a broader global trend as marriage migration is highly gendered and most marriage migrants from South-East Asia are female [8, 9]. At a global level marriage migration, where a foreign spouse migrates due to a marriage with a citizen, makes up over 10% of marriages in multiple Organisation for Economic Co-operation and Development (OECD) countries including Korea, France, Spain, Germany, the United States and the Netherlands [10]. Having an intimate relationship with a local citizen or resident in their new country sets them apart from other migrants, as their spouse is often expected to act as an intercultural link to help them adjust to their new life [11].

Migrants experience a variety of acculturation stressors as they adapt to the new culture, language, and economy of their adopted country. Acculturative stress can be intense and prolonged, which can increase the risk of mental health problems including depression and anxiety [12]. However, sufficient coping can reduce the risk [12]. Marriage migrants’ roles as transnational wives set them apart from other migrant groups as they must navigate the typical stressors experienced by migrants and in addition to those associated with their cross-cultural marriage [13, 14].

Whilst moving to a new country, migrants often continue to live transnational lives as they maintain social ties with their family and friends in their home country. This dynamic, engagement with the arrival country and maintenance of cultural ties, provides the opportunity to essentially live in two spaces. Thai migrant women often have difficulty finding suitable and long-term full time employment in Norway and this may lead some women to draw upon their networks to establish their own businesses and create their own employment opportunity [15, 16]. Massage shops and restaurants, owned by Thai migrants, are locales where women navigate sexualized stereotypes about Thai women, develop strategies to maintain their own safety and vie to be acknowledged as legitimate business persons [17, 18].

Despite the large number of Thai migrant women across the globe, there remains a paucity of literature on their mental health. In Norway, one study found that Thai migrant women were less likely than Norwegians to get conversational therapy for mental health concerns and had lower use of antidepressants [19]. However, Thai women who used an interpreter were more likely to receive counselling and specialist referral than those who did not [19]. In another qualitative study of Thai migrant women we found that some women can struggle to juggle the demands of their life in Norway and their roles as breadwinners, wives, and mothers with children in Thailand which can have a negative effect on health and wellbeing [16]. A non-governmental organisation (NGO) report found that Thai women are among the highest overnight migrant users of emergency shelters, which implies that Intimate Partner Violence (IPV) may be a challenge [20].

From neighboring Sweden, a study with Thai migrant wives reported women’s experiences of physical, psychological and economic IPV and identified that unequal power relations in Thai international marriages leave women vulnerable [21]. Another Swedish study found that exposure to IPV among Thai women was associated with poor mental health [22]. Social trust and lack of social isolation were identified as protective factors from negative health outcomes associated with IPV [22]. A study from Australia among Thai women migrants found that most mental health concerns, including stress and loneliness, were related to family difficulties spanning from communication difficulties and economic abuse to domestic violence [23]. Participants also reported stressful workplace experiences which affected their mental wellbeing [23]. In seeking help, a second Australian study reported that Thai women lacked information about mental health services, had limited English fluency and would often seek assistance from Thai monks or other Thai women [24].

Internationally, Buddhist religion and local Thai temples have been identified as an important resource for Thai marriage migrants to address their mental health needs and are often a site of recruitment for studies [23–26]. For studies on mental health, recruiting individuals from temples may lead to an oversampling of participants who use religion as a coping response. To gain insight into the transnational lives of women living in Norway we chose to collect firsthand accounts from women working in Thai massage shops. This locale, combined with the use of a Thai interpreter, helped us reach women working on the periphery of the mainstream Norwegian economy who have lower Norwegian literacy level as compared to participants in previous studies [16].

### Approach and objective

Broadly informed by the theoretical approaches to stress and coping put forward in psychology in this paper we examine stressors and coping responses among Thai migrant masseuses living in Oslo, Norway [27]. We used Folkman and Lazarus’ (1984) categorization of emotional and problem focused coping to help identify and group different types of coping strategies. In line with our projects’ overarching research objective, we also explore how these responses intersect access and utilization of public mental health services in Norway. We anticipate that understanding how Thai migrants experience stress, and their selected coping strategies will provide us with valuable information that can be used to develop appropriate mental health services for this migrant population [13].

## Methods

We conducted in-depth individual interviews (*N* = 14) with Thai migrant women who were working in Thai massage shops in Oslo, Norway. To participate, individuals had to have migrated to Norway, be above the age of 18, worked in a massage shop in the last year and have sufficient fluency in Thai or English. Our overarching study examined access to healthcare for migrant masseuses. Thai women are a growing immigrant population in Norway and while women who work in the massage industry may have particular barriers to care, there has been limited research to date. We began each interview by asking participants to describe their self-perceived health status. In seeking to understand their mental wellbeing as migrants in Norway, we inquired about stress experienced in their daily lives and coping strategies. We asked the three following open-ended questions: Do you experience stress in your daily life in Norway? How do you cope with this stress? Have you ever sought help for your mental health? We notified our research protocol to the Data Protection Official for Research, NSD - Norwegian Centre for Research Data (55206).

With support from a local NGO, the first author (NT) visited eleven massage shops in various areas of Oslo during the Fall of 2017. At each shop we introduced our project in Thai and invited individuals to participate. NT also presented our project during a workshop attended by women working in the massage industry. In approaching potential participants we explained that the primary purpose of our study was to generate information on healthcare access and use among migrants who work as masseuses in Oslo. We provided each potential participant with a recruitment flyer written in Thai language and later followed up with them if they indicated that they might be interested. We included all participants who wanted to participate. We utilized a minimal amount of snowball sampling as some participants recommended other participants. Depending on participants’ preferences, we scheduled interviews to either take place in the massage shops or in a private meeting room. NT conducted all of the interviews. Most (13) of the interviews took place with the assistance of a native Thai interpreter who had also provided feedback on the study design. Before each interview, we explained that participation in the research project was voluntary and collected informed verbal consent from every participant. The interpreter read the consent form in Thai to each participant. For the interview that was conducted in English the interviewer read the consent form to the participant. When participants gave verbal consent to participate the interviewer signed a copy of the consent form. Participants were offered a paper copy of the consent form in Thai language or English. Each interview took approximately one hour. The interviewer was vigilant to signs of distress and reiterated that participants could skip a question or feel free to discontinue the interview at any time. We audio recorded each session and the Thai interpreter subsequently transcribed and translated the recordings verbatim directly into English. NT and the interpreter subsequently reviewed the transcripts and discussed the meaning in specific sections as necessary. We reached data saturation, meaning that no new information was being collected, at 14 participants and discontinued recruitment.

We analyzed the data using thematic analysis by identifying and grouping relevant themes and organized the information in NVivo (version 11). The first author coded all of the data for deductive and emergent themes and discussed the coding with the second and fourth authors. Subsequently, the first two authors further developed the emergent themes. We utilized Folkman and Lazarus’ high level categories of emotion and problem focused coping to organize our results. To maintain participants’ anonymity, we use pseudonyms for names throughout the article.

## Results

### Description of the study sample

Of the 14 participants, more than half of the women came from rural Thailand, and many were entrepreneurs before migrating abroad. All participants had initially come to Europe because of an intimate relationship with a male citizen in their destination country and several migrated to other European countries as marriage migrants before coming to Norway. At the time of the interviews, most of the participants were divorced or separated and 40 years of age or older (Table 1). Participants had been in Norway for varying amounts of time, from less than two to over twenty years. Education levels differed, almost half were grade 9 or below (6), 5 completed high school and 3 had gone to college or to finish an undergraduate degree. Participants largely reported limited or beginner fluency in Norwegian language (10). All had worked as a Thai masseuse in Oslo, Norway in the last twelve months. Half of the participants owned the shop in which they worked. Income varied with half of the participants earning under 2400 USD (21,000 NOK) a month before taxes which is under the EU threshold for social deprivation in Norway [28].

### Stress

When asked to describe their health, four of our participants reported current or past poor mental health. However, most indicated that they experienced some type of stress in their daily lives. For our participants stress in their daily lives revolved around four key themes: settling in, loneliness, finances and spousal relationships.

#### Settling in

Upon arriving in Norway women experienced difficulty navigating the regulatory and administrative tasks necessary for employment and operating a business. Anong (49, cohabitating) explained, “First when I arrived here, I was stressed because I could not understand what people said. I was stressed with the complicated documentation when I tried to open my massage parlor. I was just able (initially) to open a personal bank account here. It is very difficult”. Participants felt initially overwhelmed by the administrative tasks associated with running a business in Norway and their challenges were compounded by language challenges and a need to independently learn how to complete business paperwork. Nin, (30s, widowed) described:

*When I arrived here, I had to learn to do everything by myself. Learning to do the paperwork by myself. My Norwegian partner did not know how to do anything. He knows Norwegian language but he did not know how to do the paperwork.*

#### Loneliness

Loneliness went beyond being away from family and friends or general homesickness and seemed to relate to the differences between a collectivist and individualist society. Norwegian society is less collectivist than Thailand and participants commented on the differences. One participant had previously lived in another Asian country with a collectivist society and found the cultural difference to be further between Norway and Thailand than the other country and Thailand. Nin, (30s, widowed) described, “I am living alone. The society does not care for others that much, unlike Thailand. I need to adapt myself a lot to this society”.

Kanok (44, single) reflected on the psychological difficulties that other women face who live alone without family members and the associated loneliness:

*Their faces have a lot more stress than mine. In my case, I could turn to my daughter, we could go out together, eat together and do family activities together. A lot of Thais will face mental issues during Christmas, as people usually spend time with their families. For people who are alone, that day is the saddest day.*

#### Finances

Financial stress was often described by participants as multifactorial, with Oslo’s high cost of living and difficulties generating sufficient income operating synergistically together. Massage shop owners could not predict their monthly income and had stress associated with this uncertainty. Ying (50s, divorced) expressed:

*Stress, yes I’m stressed nearly every day. Every end of the month I’m stressed because there’s not enough money to pay out. That is my only stress. If I had enough money, or I had somebody to help me a little then I would not be stressed. You know you have to borrow money all the time, it’s not good.*

Overall, being separated or divorced from one’s partner heightened participants financial stress as they had to pay all of the household bills independently. Kanok (40s, single) explained:

*The type of stress I cope with now is related to my recent break up with my Norwegian boyfriend. Now I just live with my daughter. I run a Thai massage parlor, I do not have a fixed income. Today I have income, but I might not have tomorrow. My life is like a balloon. You do not (know) when it is going to break. This is my stress.*

Many of the entrepreneurs who ran their own massage shops reported some financial stress due to the uncertainty of income and the high cost of rent and other expenses in Oslo. Cash flow was highly dependent on clients coming for a massage and not always predictable which left the owners stressed when business was slow.

#### Spousal relationships

Many participants reported that spousal relationships, both during the marriage and break up, were their largest source of stress in their lives in Norway. Over half of participants indicated that they had experienced some type of stress related to their relationships. While married, IPV, excess drinking, and unacceptance of the woman’s children from previous marriages were sources of conflict. A few experienced some type of violence in their relationships, be it economic, psychological or sexual, which had a significant impact on wellbeing. Waan (50s, divorced) said:

*They just pay for their beers, men here drink heavily. They also physically hit women too. I had to go to the police station. Many other Thai women here also face the same physically abusive experience, a lot. I even went to stay at a women’s shelter. See this was my life.*

Excess drinking among spouses led to arguments and put a strain on relationships. Kanok (40s, single) illustrated, “Many of them (men) have problems with excessive drinking. That is the most common reason why Thai ladies decide to break up a relationship”.

Cross-cultural disagreements about parenting and unacceptance of children from previous relationships caused stress. Waan (50s, divorced) explained:

*Another problem for Thai women who are married to a Norwegian man is that if she had children from a previous marriage, they will have problems understanding each other. The cultural and social background is different, like we are from Thailand”.*

Together IPV, excess alcohol consumption and unacceptance of a Thai woman’s children contributed to the dissolution of marriages.

Participants identified relationship break ups as a particularly stressful time in their lives. Women shared their accounts of being left for another woman, the betrayal and the associated financial implications. Ubon (40s, divorced) said:

*They knew each other from there (my work place) …*. *When I returned from Thailand, I was not allowed to go into the house that we used to live in together …*. *My ex-husband in the past he helped me financially but when he had a new woman, he stopped giving me money.*

Getting pushed out of one’s home not only occurred during a break up. Beyond Ubon, another two participants described getting kicked out of their house by their ex-husband after an argument, leaving them without a place to stay. Madee (40s, divorced) previously lived in rural Norway expressed:

*The city is fine and good. But for people in the countryside, here rural is very rural. From my own experience around midnight or one am in the morning my husband kicked me out from the house. I did not (know) where to go and get help at that time. In the city it is easy, you go out and there are people, but in the countryside you go out and there is no one.*

One participant reported custody difficulties after being kicked out of the house and was pursuing legal avenues to be able to have her child live with her. While break ups were identified as stressful events, they also contributed to more financial stress. Unequal control over assets amplified the negative financial impact of break ups on women. These assets include housing and businesses. Waan (50s, divorced) described her situation when she broke up with her husband:

*It is the house that I first moved into when I married him. On the date that we broke off our relationship, he told me that he would not give me anything as I came here with nothing. What we bought together, we both helped to pay, which was not a problem for me. I told him that I wanted freedom, not the money*.

Another woman who had set up a business with a male partner in another European country, lost everything when the relationship ended as the business was in her partner’s name.

### Coping strategies

In addressing mental health stressors, participants described taking two broad approaches: emotion-focused coping and changing the situation through problem focused coping.

#### Emotion-focused coping

Emotion-focused coping, including both constructive and detrimental methods, was the most widely adopted approach among participants [29]. In emotion-focused coping individuals use their own resources to deal with stress by modulating their emotional response [27]. Women sought to self-cope through avoidant coping strategies such as distraction or drinking and active coping strategies utilizing Thai lifestyle, cognitive thinking and Buddhist philosophy.

To distract themselves from stress, participants purchased plants to beautify their environment, watched Thai TV programs, listened to music and sang Karaoke. Ying (50s, divorced) explained, “Watch TV a little bit to make you forget everything. Watch a movie, a funny thing or something like that”.

A couple of participants used drinking as a detrimental coping strategy to give temporary relief from dealing with spousal conflicts. Ubon (40s, divorced) drank until passing out due to financial problems in Thailand and a marital break up in Norway which included getting kicked out of her house. She explains, “Sometimes, I still have a problem with drinking. I drink wine, one glass, two glasses, and could not stop. I drink the whole bottle”.

Embracing Thai cultural practices was an active way participants coped with stress. For many this meant spending time with their children and grandchildren thereby passing on Thai cultural values. Waan (50s, divorced) expressed, “Now I am happy with my granddaughter … She likes to talk a lot. She says thank you in Thai”.

Participants described living as though they were in Thailand as a strategy to reduce their daily stress. This Thai lifestyle approach for two of the older participants is linked to practicing meditation and using Buddhist philosophy to cognitively reframe their situation. They described this as being conscious. Waan (50s, divorced), “I just have to make myself feel good and do not need to worry (about) anything. They told me humans are born by ourselves and will go by ourselves as well one day”. This approach brought them piece of mind. Lamai (50s, divorced) explains “My life is fulfilled, I am living like I am in Thailand”. Cognitive thinking was also identified as a strategy to address future stress. Benja (40s, single) said, “In the future if I encounter stress, I have to be conscious. It is the first thing to do, right? We then have to review the causes for the stress”. Cognitively reframing their life situation as a coping strategy lead to self reliance.

Participants emphasized accepting their situation, being strong and working independently to solve their own problems. When dealing with financial difficulties, Ying (50s, divorced) expressed, “Cannot do anything. Just gonna have to live like this”. Benja (40s, single) explained that Thai women facing marriage dissolution should be independent, “You have to stand on your own feet”. Women felt responsible for taking care of their own problems and mental health. Ubon (40s, divorced) expressed, “I have to take care of my own mind, I could not just rely on doctors. I will just get the doctors’ time”.

Participants did not want to burden their family members in Thailand with their stress, as to not cause worry. Kanok (40s, single), “I do not want to call my dad (living in Thailand) to express my stress, he is 96 years old. I do not want my family to worry”.

In some cases, in additional to their emotion-focused self-coping women were also providing psychosocial support to other Thai women to help them deal with their stress. Ubon explained, “I gave them advice (other Thai women). When I face my own problems, I have to solve it too”.

#### Changing the situation through problem based coping

Changing their situation was another coping strategy that women used to deal with stressors. Ultimately, many of the participants left their partner and in several cases they moved from another EU country to Norway to get space from their ex-partner and begin a new life. Janthana (30s, divorced) explained, “I love Oslo because everything is new. My brain feels lighter. I am an open person. I felt that I wanted to move to live in a new country. Every place I went (in another European country) I had been there with my ex-partner”.

Two participants used the Norwegian legal system. Madee (40s, divorced) who was raped by her husband talked about using the legal process to help move past her experience, “I’m passed it now, it is done in the court. Thai ladies when they come and face problems like me, they do not fight back and just accept it. As I am here, I want all Thai ladies facing the same situation to combat”. Another participant went to court to seek custody of her son.

While often seeking to solve their stress alone, some women sought external help from friends and family members, social workers and medical professionals. Women spoke with their Thai friends to reduce their stress and to seek practical advice. Visits with family also helped to reduce stress. Anong (40s) who was cohabitating, indicated that her partner was a source of support, “My Norwegian partner calms me down. He will help me with everything”.

Several participants sought help from Anne (pseudonym), a Norwegian social worker who is fluent in Thai for assistance with translation, and navigation of Norway’s healthcare and legal systems. Madee (40s, divorced) described:

*I called Anne. I am fortunate to have Anne’s business card. I could not talk with the psychologist due to the language problem. I did not go to the Thai temple. I was helped by Anne. She took me to the doctors. The doctors saw me and they gave me a prescription for stress relief medications to take before going to sleep.*

Two participants independently sought assistance from their Norwegian general practitioner, the first for sleeping pills and the second received a referral to a psychologist. The woman who went to see the psychologist had been living in Norway for 18 years and spoke to the healthcare provider in Norwegian.

Two other women wanted to be able to see a psychologist but were not sure where to begin. Limited linguistic capacity in Norwegian was also identified as a barrier to health system navigation and accessing mental health care. In addition, Nin (30s, widow) described the Thai stigma surrounding mental health and seeking professional help:

*I think some people want to see psychologists but they are not brave enough to go. It’s like me, sometimes I think that I am so stressed out, it is like I am becoming crazy. But I am not able to talk with anyone. They are not brave, they are afraid of someone else looking at them negatively. For Thai people, seeing a psychologist is for someone who has completely lost their mind. But I do not think the same as Thai people living here because I faced mental health issues from my own experience. I was living with someone who had abnormal psychological conditions. Sometimes, I felt like I wanted to share my feelings with someone but they would not be able to accept it. Sometimes I want to see a psychologist to find out what has happened to me.*

Nin was supporting her partner who had mental health challenges and it also started to affect her own mental wellbeing but she didn’t know where she could get support.

## Discussion

### Sources of stress

Overarchingly, our results that migrant masseuses experience stress due to administrative tasks, loneliness, financial difficulties and intimate relationships, corroborate study findings from both Norway and other countries [14, 16, 23]. Participants’ reports of stress as they transition to living in Norway and complete the necessary administrative tasks are consistent with the international literature that suggests migrants experience acculturation stress when relocating to a new country [3]. Our finding that finances, primarily related to owning a business, are a source of stress is not surprising given the high cost of living in Oslo as compared to the salary of masseuses. Remittances are often mentioned as a source of relationship stress in studies on marriage migrants, however this was reported by few participants in our study which may suggest that it is not always conceptualized as a stressor in daily life by migrants themselves [16, 30, 31].

Being engaged in a social network is important for Thai women and not being actively involved in the new society may be stressful for this group. Theoretically, as the social environment shifts from collectivist to more individualist, migrants from collectivists societies may experience greater stress in their daily lives [32]. One of our participants, for example, explained the difference between living in another Asian country and Norway. Mental health professionals in Norway should keep this in mind when counseling Thai migrants. The level of social engagement required for good wellbeing may be very different for Thais and Norwegians. Social isolation has been associated with poor mental health in other studies of Thai migrants, however few studies have looked at differences based on the social organization of receiving societies and more research may be warranted in this area [22, 23].

Half of our interview participants were entrepreneurs. As business owners they assumed financial risk and associated financial stress. A study of Thai entrepreneurs in Sweden, reported that while having a Swedish husband provided Thai women with support to navigate the administrative tasks necessary to establish a business in Sweden, it could cause difficulties if they left their husband as business and marriage were closely intertwined [18]. Our data supports this nuance, as women often looked to partners for support when establishing businesses but in one instance a woman was left vulnerable when the relationship ended and the entity was registered in their partner’s name. This may suggest that Thai migrants may need more assistance in establishing their own businesses so they enjoy greater financial autonomy.

Spousal relationships were cited by participants as the biggest source of stress in their lives. Family difficulties, as a primary source of stress, was also reported in Australia among Thai female migrants [23]. Another Swedish study found that relationship breakups were stressful for Thai migrant women [21]. In our study, this finding is interesting from a temporal perspective as we asked participants about stress experience in their daily life and at the time of the interviews most were already separated or divorced. It is possible that participants may be still working through the stress associated with these events in addition to the associated financial difficulties which continue to affect them after the dissolution [33].

A number of our participants experienced IPV, however we don’t currently have any statistics for IPV among Thai marriage migrants in Norway. Our qualitative results support the assertion from neighboring Sweden that those who experience IPV have poorer mental health than other Thai migrants [34].

### Coping strategies

When faced with daily stressors, women often used “Thainess” as an overarching strategy to cope with stress. The term “Thainess” is frequently used in political science and South East Asian studies as a description of Thai identity [35]. The term is often defined in opposition of the other, or that which is not Thai. In this article we use this term to be inclusive of both identity and associated cultural practices. “Thainess”, which we define as immersing oneself in Thai cultural practices including the use of Buddhist cognitive thinking, appears to be a culturally specific response which emphasizes self-reliance as well as supporting other Thai women. Elements of this approach, specifically Buddhist techniques and support from Thai friends have been observed among Thai women in other countries, however our study is the first to describe this as a coping strategy [23]. Participants talked about living as though they were in Thailand to alleviate stress and it appears that they use this approach to adapt to their local environment while maintaining their mental wellbeing.

In navigating stress, participants did not want to worry their parents in Thailand and thus were silent about their difficulties. Not wanting to burden family contributes to a silence surrounding difficulties experienced by marriage migrants and helps sustain positive connotations in Thailand associated with marriage migration to Europe. This silence has been documented in other studies of Thai migrants and we anticipate that it ultimately helps sustain migration flows [16, 31].

Once the situation became untenable women took action, by moving to a new country, going to court or confronting partner. Moving to a new host country, as a coping strategy for relationship stress has not been widely documented in Thai marriage migrant literature. These problem-focussed approaches show women’s strength and determination to improve their situation and overcome complex obstacles.

As most of our participants were divorced or separated, it is not surprising that only one participant sought support for stress from their spouse. We observe that there is a strong network of Thai women within Norway, and many other European countries, through which women may reach out to Thai friends for psychological and logistical support [18, 25].

Although discussions during the interviews were mostly about daily stressors, prolonged levels of stress can impact mental health considerably. Indeed, several women had indicated having poor mental health, yet only two had sought professional help. In seeking mental health services from the Norwegian public health system, participants reported stigma and language as barriers. Both have been documented in Norway and other contexts and neither are specific to Thai migrants [23, 36–38]. Systems navigation may also be difficult as several women indicated a need to see a psychologist but were not sure where to begin. In addition, participants’ preference of self-coping and informal support may reduce their use of Norwegian mental health services. Preferences for self-coping and informal support have also been documented among Filipina migrants in Norway [39]. To help Thai migrant women access mental healthcare, GPs and other clinicians may wish to probe individuals about their mental wellbeing. With the support of a translator, clinicians can collect detailed case histories to better understand their Thai women patients’ sources of stress and coping strategies. This information can help GPs to recommend appropriate mental health resources.

In our study, a Norwegian social worker who is fluent in Thai played the role of an interpreter and systems navigator in helping participants get access to healthcare for mental health. Use of similar navigators have been found to be effective in increasing access to healthcare in other migrant populations and this method could be effective on a larger scale in Norway [40]. Network approaches, such as having a Thai health systems navigator who acts as a bridge between the health system and informal Thai networks, should be considered by health policy makers. System navigators can help migrants utilize existing resources within the health system and link women to additional opportunities provided by other organizations. For example, to improve geographical coverage a psychologist could be paired with an interpreter/navigator to provide e-consultation to Thai women living throughout the country. During our research we discovered that the Thai embassy provides online counseling with qualified mental health professionals in Thailand. The online counseling pilot project “Ooca-Norway” provides mental health counselling free of charge through the internet for Thais living in Norway with certified psychologists in Thailand [41]. The Ooca-Norway interface of the application and service are provided in Thai language [42]. The existence of this online counseling service and an extension of the pilot project due to popularity suggests there is a need for mental health supports for Thai people living in Norway. This online counseling project is a collaborative initiative with the Thai Women Network in Europe (TWNE), a grassroots organization that assists Thais in Europe and helps to defend their rights [43]. Organizations working with Thai migrant women in Norway and other countries should consider preferences for self-coping, mental health stigma in Thai communities, and linguistic competency when designing mental wellbeing interventions for Thai women.

### Avenues for future research

Specific to Norway there remains limited information on the mental wellbeing of Thai migrants. In addition, little is currently known about IPV among Thai marriage migrants in Norway. Based on our small sample and media reports, we anticipate that it would be valuable to conduct a survey to examine IPV and mental health as well as the services utilized by this population [44, 45].

Comparative to the Norwegian born population, participants in our study had significantly lower levels of education. We did not collect information on participants’ motivation for becoming a masseuse in Norway or integration into Norwegian society, and are unsure if they chose this career amongst a variety of options, or whether they opted to be a masseuse as they could easily access this type of job through their Thai social network. However, participants worked at Thai owned massage shops and use this network for social support, so it is possible that many are living a life set apart from other Norwegians. Further research is warranted on workforce integration among Thai marriage migrants in Norway as there are some important intersections between migration, work and health [46].

An estimated 1.8 million Thai women migrants live outside of Thailand [47]. Given the size of this population and some reports of IPV, more research on their mental wellbeing is warranted. In developing an international research agenda we propose the following questions to help guide the inquiry: Is “Thainess”, cultural norms and Buddhist philosophy, a culturally specific coping strategy for stress utilized by Thai migrants internationally? Do outreach workers who provide interpretation and health systems navigation improve Thai migrants’ uptake of mental health services? Do levels of integration into the national labour market influence Thai migrants’ mental wellbeing?

### Strengths and limitations

This is the first health study focussed on Thai masseuses in Scandinavia. By selecting a specific occupation, predominantly practiced by Thai migrants, we were also able to collect information specific to gender, work and health. Comparative to other studies on Thai female migrants in Norway, our participants had less education and a lower command of Norwegian, which may help bring forward new perspectives [16]. In addition, half of our participants were entrepreneurs which adds to the literature from neighboring countries about Thai women who are creative risk takers [17, 18, 26]. One limitation of our study is that most of the participants were divorced and separated and as such their experiences may be different from other Thai marriage migrants who are still married. In addition, our study is specific to Thai women who are working as masseuses and is not generalizable to Thai women in Norway working in other occupations.

To enhance the trustworthiness of the findings, a Thai interpreter assisted with data collection. Both the interviewer and interpreter were new to Oslo and had few social connections among Thais living in Norway, which seemed to make participants feel more comfortable in sharing their personal experiences. The first author has basic level Thai language skills and second author has completed other studies on health of Thai migrants in Norway, the third author is an expert in the Norwegian healthcare system and the fourth author provided expertise in migration and mental health, all which helped to develop a comprehensive analysis of the data.

## Conclusion

Thai massage is a culturally specific profession mostly practiced in Scandinavian countries by Thai migrant women. Massage shops are often owned by Thai women entrepreneurs and are a locale where cultural knowledge is utilized to bridge migrants into the local economy. Previously little has been reported about Thai migrant masseuses’ mental wellbeing. Participants in this study reported stress related to administrative tasks necessary to set up a small business, loneliness, finances and spousal relationships. Relationship conflicts were the largest source of stress. Migrants in our study often adopted “Thainess”, the use of Thai cultural practices and Buddhist cognitive thinking, as a self-coping strategy for stress. This is the first study to use the term “Thainess” in relation to mental wellbeing. Preferences for self-coping, mental health stigma, and limited linguistic competency should be considered when designing mental wellbeing interventions for Thai women. Clinicians, with interpreter assistance, may take detailed case histories to better understand their Thai women patients’ sources of stress, coping strategies and general wellbeing. Network approaches, such as Thai health systems navigators who act as a bridge between the health system and Thai networks, are also warranted.

## Data Availability

The data we collected contains personally identifying information. To protect participant confidentiality we cannot share the data.

## Abbreviations

IPV: Intimate Partner Violence
TWNE: Thai Women Network in Europe
